# Endovascular Treatment of Acute Ischemic Stroke in Clinical Practice: Analysis of Workflow and Outcome in a Tertiary Care Center

**DOI:** 10.3389/fneur.2021.657345

**Published:** 2021-06-07

**Authors:** Karin Weissenborn, Sam Gruber, Gerrit M. Grosse, Maria Gabriel, Ramona Schuppner, Hans Worthmann, Omar Abu-Fares, Friedrich Götz

**Affiliations:** ^1^Clinic for Neurology, Hannover Medical School, Hannover, Germany; ^2^Hannover Medical School, Institute for Diagnostic and Interventional Neuroradiology, Hannover, Germany

**Keywords:** acute ischemic stroke, mechanical recanalization, work-flow, outcome, onset-to-groin-time

## Abstract

**Background and Purpose:** Pre- and intra-hospital workflow in mechanical recanalization of large cervicocephalic arteries in patients with acute ischemic stroke still needs optimization. In this study, we analyze workflow and outcome in our routine care of stroke patients undergoing mechanical thrombectomy as a precondition for such optimization.

**Methods:** Processes of pre- and intra-hospital management, causes of treatment delay, imaging results (Alberta Stroke Program Early Computed Tomography Score, localization of vessel occlusion), recanalization (modified thrombolysis in cerebral infarction score), and patient outcome (modified Rankin scale at discharge and at the end of inpatient rehabilitation) were analyzed for all patients who underwent mechanical thrombectomy between April 1, 2016, and September 30, 2018, at our site.

**Results:** Finally, data of 282 patients were considered, of whom 150 (53%) had been referred from external hospitals. Recanalization success and patient outcome were similar to randomized controlled thrombectomy studies and registries. Delay in treatment occurred when medical treatment of a hypertensive crisis, epileptic fits, vomiting, or agitation was mandatory but also due to missing prenotification of the hospital emergency staff by the rescue service, multiple mode or repeated brain imaging, and transfer from another hospital. Even transfer from external hospitals located within a 10-km radius of our endovascular treatment center led to a median increase of the onset-to-groin time of ~60 min.

**Conclusion:** The analysis revealed several starting points for an improvement in the workflow of thrombectomy in our center. Analyses of workflow and treatment results should be carried out regularly to identify the potential for optimization of operational procedures and selection criteria for patients who could benefit from endovascular treatment.

**Subject terms:** ischemic stroke, interventional stroke therapy, quality and outcome.

## Introduction

The implementation of intravenous thrombolysis (IVT) and, more recently, mechanical recanalization of occluded large intracranial arteries into stroke therapy tremendously improved the outcome of acute ischemic stroke (1–8). As with IVT, indications and contraindications for endovascular treatment (EVT) have been continuously challenged and tailored to the patients' demands. The time window for EVT has been extended up to 24 h in patients with a mismatch between clinical deficit and infarct or with a mismatch between ischemic and infarcted tissue (9, 10), and the criteria for eligibility for EVT were expanded to patients with Alberta Stroke Program Early Computed Tomography Score (ASPECTS) <6 and National Institutes of Health Stroke Scale (NIHSS) score <6 (5, 10–14). There are continuous efforts to improve patient selection for thrombectomy and to optimize pre- and intra-hospital procedures. By thorough workflow analysis, weak points in the treatment process can be detected and eliminated, thereby facilitating satisfying treatment results.

We report on the workflow analysis and treatment results of all stroke patients who received mechanical thrombectomy at our tertiary center between April 1, 2016, and September 30, 2018.

## Methods

All consecutive patients with acute ischemic stroke and large vessel occlusion (LVO) who underwent EVT at our site were prospectively enrolled into a local registry. Institutional review board approval was obtained for a retrospective review of these prospectively collected data in a quality assurance database for which consent was waived. Data were prospectively collected by SG, who interviewed the attending physicians within 24 h after the procedure, if possible. In addition, the emergency room (ER) neurologists were requested to document any observed cause of delay in their procedure reports. The indication for EVT was established in consensus between the attending neurologist and the interventional neuroradiologist on a case-by-case basis considering all available clinical and imaging data. Patients were either directly admitted to our hospital or transferred from external hospitals. Intravenous thrombolysis was indicated and applied according to national guidelines (15).

### Clinical Data

Age, sex, family status, health insurance, time of symptom onset or last known well, clinical symptoms, Trial of Org 10172 in Acute Stroke Treatment classification, NIHSS at admission, use of anticoagulants or platelet inhibitors before EVT, and cerebrovascular risk factors were documented.

### Radiological Data

Intracranial hemorrhage was excluded by cranial computed tomography (CCT) or—especially in the case of unknown time window since stroke onset—magnetic resonance imaging (MRI). LVO was proven by CT angiography (CTA) or magnetic resonance angiography (MRA) and confirmed *via* conventional angiography. ASPECTS and posterior circulation ASPECTS (16) were used to classify the extent of infarction in the CCT or MRIs on admission.

### Procedural Data

A team of seven interventional neuroradiologists was available for the EVT on a 24/7 schedule at our site (from 5.00 p.m. to 8.00 a.m. as on-call service). Aspiration catheters, stent retrievers, or both were used on a case-by-case basis. The additional use of intra-arterial thrombolysis, anticoagulants or antiplatelets, and stenting of the occluded or stenotic vessel or the connected upstream vessel was at the discretion of the operator as well. EVT was preferentially done under general anesthesia. Symptom onset-to-groin puncture time, onset-to-needle time, door-to-imaging time, door-to-needle time, door-to-groin time, and onset- and door-to-recanalization time were documented for further analysis as were any circumstances that delayed treatment from the attending neurologist's view (such as delirium, extended vomiting, or severe hypertension demanding treatment before EVT or unavailability of the imaging facilities, for example). “Door time” refers to the time of admission to our center.

Workflow during standardized working hours was compared with that during on-call shifts, as well as the workflow on working days to that on weekends. Additionally, the number of patients seeking neurological treatment at the ER within ±1 h of the arrival of the EVT patients was documented, as was the number of neurologists present at the ER when the patient arrived.

### Outcome

Short-term clinical outcome was assessed by NIHSS and modified Rankin scale (mRS) at discharge, long-term outcome by mRS at discharge from the rehabilitation clinic. The radiological outcome was assessed by the modified thrombolysis in cerebral infarction score (mTICI) (17); mTICI scores 2b and 3 were considered favorable. In addition, the need for decompressive hemicraniectomy and occurrence of secondary intracranial hemorrhage were recorded.

### Statistical Analysis

Statistical analysis was done using IBM SPSS Statistics 26 (SPSS Inc., Chicago, IL., USA). Normally distributed continuous data were described as mean ± standard deviation and non-normally distributed continuous variables as median with the 25th and 75th percentile. A Gaussian distribution was verified by using the Kolmogorov–Smirnov test. Comparison of non-normally distributed continuous variables was done using the Mann–Whitney *U*-test. Categorical data were analyzed by using the chi-square test. Correlations were analyzed with the Spearman rho test. Risk factors for an unfortunate outcome (mRS 3–5 and mRS 6) were determined by multinomial logistic regression analysis, including all parameters that had been shown to differ significantly between these patient groups and those patients with good outcomes (mRS 0–2). The significance level was set at *p* < 0.05.

## Results

### Baseline Characteristics

**Figure 1:**
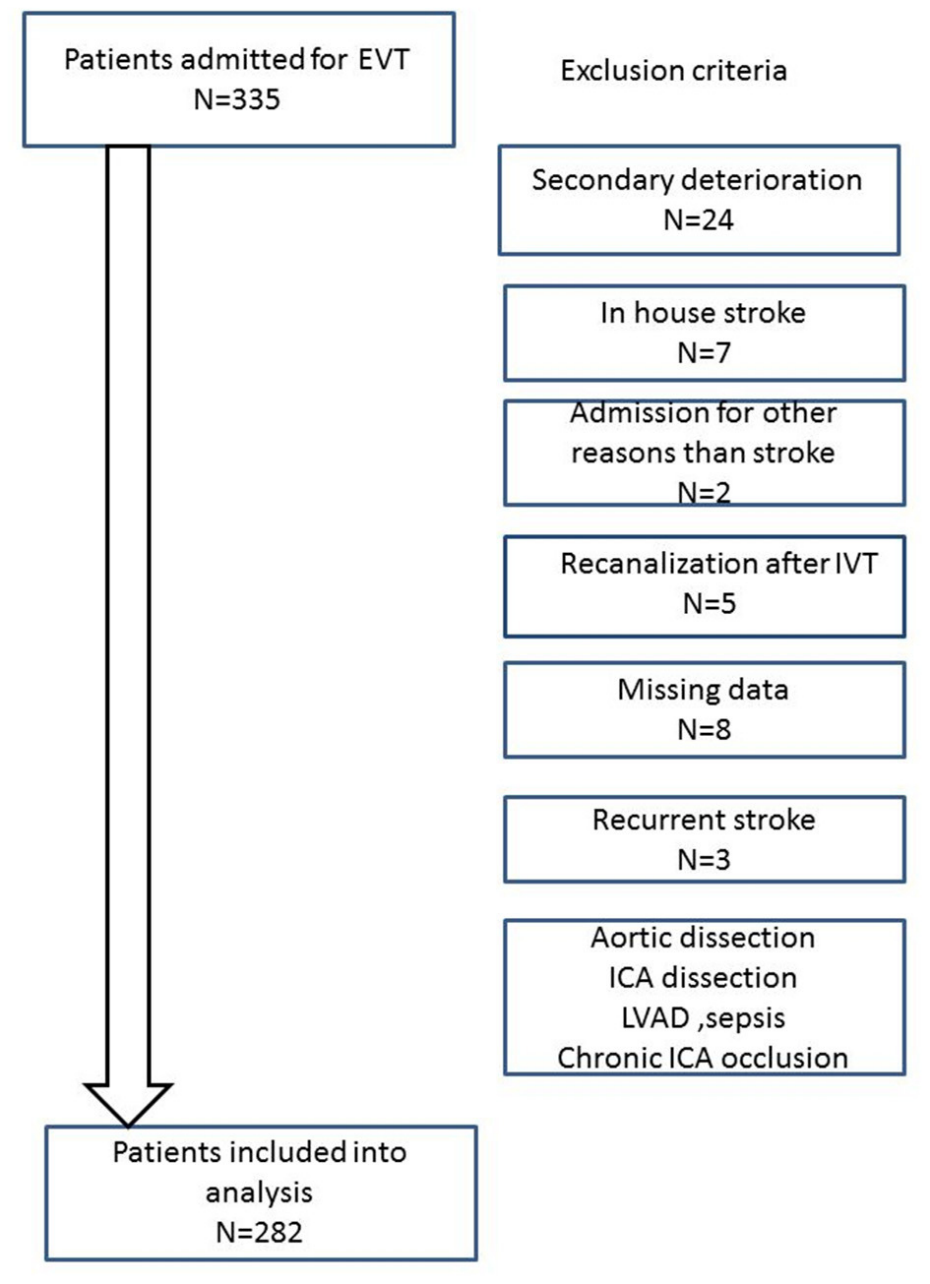
Flowchart displaying the exclusion criteria. Patients with secondary deterioration had presented without neurological symptoms or a rapid improvement in the emergency room but had developed severe neurological deficits hours later. As we aimed to analyze the quality of our prehospital care and flow of our emergency processes, we decided to exclude these patients from further analyses because they are not representative of the “usual” workflow.

From April 1, 2016, until September 30, 2018, 335 patients with acute ischemic stroke underwent EVT at our institution. Fifty-three patients were excluded from further analysis for various reasons. Details are given in Figure 1. A total of 132 of the remaining patients were directly admitted to our hospital, whereas 150 were transferred from external hospitals for further treatment from a distance of up to 100 km. Clinical and imaging baseline characteristics of the included patients can be found in Tables 1, 2.

**Table 1:** Baseline characteristics of patients directly admitted to MHH vs. patients referred from external hospitals.

	**Patients directly admitted to MHH *N* = 132**	**Patients referred from external hospital *N* = 150**	***p*-value**
Age (years)	77 (40–94)	75 (23–91)	0.03
Sex (m/f)	59/73	89/61	0.01
Family status single/married/unknown	55/70/7	52/89/9	0.48
Statutory/private health insurance	105/27	114/36	0.45
NIHSS on admission	15 (2–40)	15 (1–40)	0.85
Symptom onset unknown	62/132 (47.0%)	53/150 (35.3%)	0.05
Intravenous thrombolysis	104/132 (78.8 %)	100/150 (66.7 %)	0.08
Diabetes mellitus	37/132 (28.0%)	35/150 (23.3 %)	0.37
Hypertension	92/132 (69.7%)	117/150 (78.0%)	0.11
Hypercholesterolemia	64/132 (48.5%)	73/150 (48.7%)	1.00
Atrial fibrillation	74/132 (56.1%)	82/150 (54.7%)	0.15
Platelet inhibitors	39/132 (29.5%)	42/150 (28.0%)	0.78
Vitamin K antagonist	19/132 (14.4%)	18/150 (12.0%)	0.55
DOAC/LMWH	13/132 (9.8%)	27/150 (18.0%)	0.05
TOAST classification			0.35
Large artery atherosclerosis	11/132 (8.3%)	15/150 (10.0%)	
Cardioembolic	74/132 (56.1%)	78/150 (52.0%)	
Other etiology	7/132 (5.3%)	3/150 (2.0%)	
Unknown etiology	40/132 (30.3%)	54/150 (36.0%)	

*M, male; f, female; NIHSS, National Institute of Health Stroke Scale; DOAC, direct oral anticoagulants; LMWH, low molecular weight heparin; TOAST, Trial of Org 10172 in Acute Stroke Treatment*.

**Table 2:** Neuroradiological data.

	**Patients directly admitted** ***N* = 132**	**Patients referred from external hospital** ***N* = 150**	***p*-value**
**Occlusion site**			
ICA	19 (14.4%)	9 (6.0%)	0.10
ICA + MCA	25 (18.9%)	32 (21.3%)	
MCA	76 (57.6%)	85 (56.7%)	
BA	12 (9.1%)	22 (14.7%)	
VA	0	2 (1.3%)	
ASPECTS on admission	7 (6/9) (*n* = 116)	6 (5/8) (*n* = 111)	0.220
pc ASPECTS on admission	8 (7/10) (*n* = 13)	8 (6/9) (*n* = 23)	0.371
**Technical details of EVT**			
Aspiration catheter	43/132	50/150	0.37
Aspiration + stent retriever	68/132	85/150	
Intracranial stent	1/132	3/150	
No thrombotic material retrievable	5/132	3/150	
Site of occlusion not accessible	15/132	9/150	
Extracranial stent	23/132 (17.4 %)	35/150 (23.3 %)	0.22
**Processing times**			
Door-to-imaging time (min)	18 (13/24)	17 (11/23) (*n* = 67)	0.24
Door-to-groin time (min)	81 (64/105)	42 (28.75/68) Without further imaging (*n* = 83): 30 (25/39) Additional CCT (*n* = 34): 54 (42.5/66.25) Additional MRI (*n* = 28): 81 (65/104) Additional CCT + MRI (*n* = 5): 134 (91.5/225)	<0.001
Onset-to-groin-time (min)	145 (114.75/174) (*n* = 70)	255 (206/313) (*n* = 99)	<0.001
Groin-puncture-to-recanalization (min)	76 (45/102)	84.50 (50/120.5)	0.17
Onset-to-recanalization (min)	221 (187.25/277) (*n* = 70)	342 (293/435) (*n* = 99)	<0.001
Door-to-recanalization (min)	156 (127.25/205)	128.50 (94.25/179.25)	<0.001
**Recanalization**			
mTICI 0	25 (18.9%)	22 (14.7%)	0.70
mTICI 1	3 (2.7%)	5 (3.3%)	
mTICI 2a	8 (6.1%)	14 (9.3%)	
mTICI 2b	52 (39.4%)	56 (37.3%)	
mTICI 3	44 (33.3%)	53 (35.3%)	

*ICA, internal carotid artery; MCA, middle cerebral artery; BA, basilar artery; VA, vertebral artery; ASPECTS, Alberta Stroke Program Early CT Score; pc, posterior circulation; EVT, endovascular treatment; min, minutes; CCT, cranial computed tomography; MRI, magnetic resonance imaging; mTICI, modified thrombolysis in cerebral infarction score*.

Transferred patients were younger and more frequently men. Moreover, the time of symptom onset was more often known in this patient group.

The majority of the patients were admitted during standby service (185 of 282; 65.6%) with an equal distribution regarding weekdays. A total of 110 of the 132 patients (83.3%) who were directly admitted were announced in advance by the emergency medical staff.

Initial imaging was CCT and CTA in 93 (70.4%), MRI and MRA in 22 (16.7%), and CCT plus MRI in 17 (12.9%) of the 132 patients who were directly admitted to our hospital compared with 100% of CCT/CTA in the transferred patients. The distribution of occlusion sites and ASPECTS was similar in the directly admitted and the transferred patients (Table 2), as was the stroke etiology according to the Trial of Org 10172 in Acute Stroke Treatment classification (Table 1). In 204 cases (72.3%), EVT was accompanied by IVT.

### Endovascular Treatment

EVT was performed under general anesthesia in 261 (92.6%) patients and under conscious sedation in 21 (7.4%). An aspiration catheter was used more often in conjunction with a stent retriever (153 patients) than exclusively (93 patients). Intracranial stents were placed in four patients. In 24 patients (8.5%), EVT was not successful due to technical obstacles such as significantly tortuous vessels precluding access to the thrombus or advanced atherosclerosis of the femoral artery precluding arterial access. In 13 patients, rt-PA (recombinant tissue plasminogen activator) (between 5 and 20 mg) was administered intra-arterially during the mechanical recanalization procedure. In 58 patients with tandem occlusions, stenting of the cervical occluded or stenotic vessel was performed in addition to recanalization of the intracranial branch to secure adequate blood flow to the affected area. The door-to-groin time was 64 min in median (25th/75th percentile: 40.0/91.7 min). The EVT procedure lasted 80 min (25th/75th percentile: 47.5/115 min). The onset-to-groin time was 205 min (25th/75th percentile: 149.0/272.5 min) and the onset-to-recanalization time 300 min (25th/75th percentile: 220.0/365.5 min) in those with known symptom onset. Satisfactory recanalization (TICI 2b/3) was achieved in 205 of 282 patients (72.7%).

**Figure 2:**
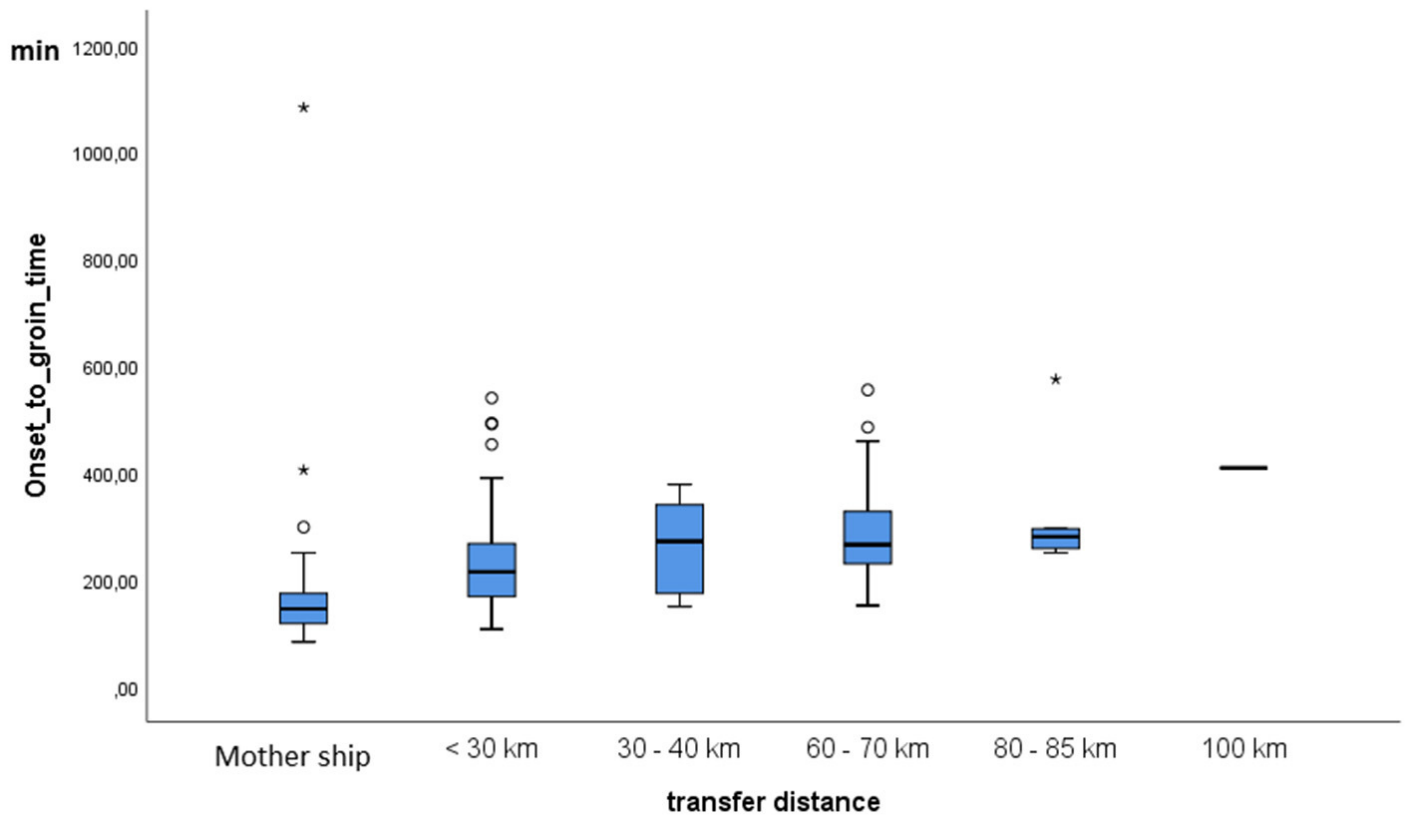
Onset-to-groin time in dependence of transfer distance. MHH, Hannover Medical School. *Represents extreme outliers in the distribution.

Table 2 compares the respective data of the directly admitted to the transferred patients. The need for transfer increased the onset-to-groin time by more than 100 min in median, mainly depending on the distance between the hospitals. However, even transport between hospitals within Hannover delayed groin puncture by ~60 min (Figure 2), and deficits in transfer organization were recorded in 28 patients who came from external hospitals (18.7%) but also in 13 of 132 cases (9.9%) who were directly admitted.

### Causes for Delay of Endovascular Treatment

Several causes of delay were reported, some avoidable, others inevitable. Brain imaging was delayed in 13 cases due to unavailability of CCT or MRI, preference of MRI, or request of both MRI and CCT by the interventional neuroradiologist. In eight patients, a concurrent intervention delayed the start of EVT. In 67 transferred patients, the interventional neuroradiologist on duty requested additional brain imaging (CCT in 34, MRI in 28, both in 5) to confirm the indication for EVT, usually because the transfer had taken a significant amount of time. The duration of this additional imaging is outlined in Table 2. Further reasons for the delay of EVT were lack of a venous line on admission (*n* = 17), delay in ER procedures (*n* = 17), severe hypertension (*n* = 16), interdisciplinary discussion about the indication for EVT in borderline cases (*n* = 22), agitation, seizures or vomiting (*n* = 25), extensive information of patients or relatives (*n* = 16), non-availability of an anesthesiologist (*n* = 14), deficits in communication between departments (*n* = 8), primary admission to another specialty than neurology (neurosurgery, trauma surgery; *n* = 5), and technical problems such as malfunction of the CCT (*n* = 6).

A multiple linear regression analysis using door-to-groin time as the dependent variable and sex, age, family status, admission during regular working hours, NIHSS on admission, ASPECTS on admission, number of delays, and transfer for EVT as independent variables showed that admission outside of working hours (B: −19.29, CI: −28.63 to −9.94) and transfer for EVT (B: −29.64, CI: −38.6 to −20.67) significantly decreased the door-to-groin times, whereas with every single cause for delay noticed, the door-to-groin time increased by 7 min (CI: 4.3 to 11.11 min).

The number of patients who attended the neurological ER within 1 h before and after the admission of the EVT patient had no impact upon the door-to-groin time. However, the door-to-groin time was shorter if the ER was staffed with two neurologists instead of one (median 60 vs. 70 min, *p* = 0.037).

The number of interventions performed by the individual neuroradiologists during the observation period differed notably (between 27 and 110), as did their median door-to-groin time (49.5–89.0 min; *p* < 0.001). The door-to-groin time decreased with increasing experience of the interventionalist regarding EVT.

### Outcome

The NIHSS at discharge was 12 (25th/75th percentile: 3.0/20.25) and was not significantly different between directly admitted (median NIHSS 12.0; 25th/75th percentile: 3.0/21.0) and transferred patients (median NIHSS: 11.0; 25th and 75th percentile: 3.0/18.2) (*p* = 0.77). Median mRS at discharge was 5 for all patients, as well as for the two subgroups (25th/75th percentile: 2/5). Only 79 of the 282 patients (28%) achieved a mRS of 0–2, of those with ASPECTS ≥ 6, 33.4%. There was no difference between the directly admitted and transferred patients. Sixty-one patients died in hospital (21.6%): 27 (9.6 %) received palliative care following the demands of the patient's provision, eight patients each died due to space-occupying intracranial hemorrhage or brain stem infarction, five from malignant MCA infarction, five from sepsis, four from aspiration pneumonia, and four from preexisting severe accompanying disease. There was no difference between the two patient groups.

**Figure 3:**
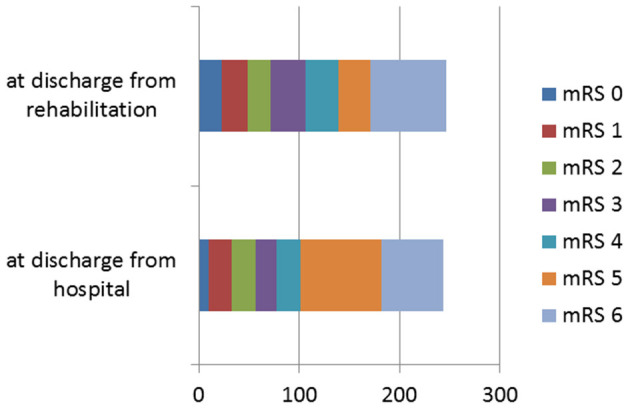
Modified Rankin Scale (mRS) at discharge from hospital and after inpatient rehabilitation for *n* = 246 patients for whom data at both time points were available.

mRS at discharge from rehabilitation (in median at 68.5 days; 25th/75th percentile: 43.7/90 days) was available for 246 patients. Fourteen additional patients had died, increasing mortality for this subgroup from 24.8 to 30.5%. However, the number of patients with mRS 0–2 increased from 57 (23.2%) to 71 (28.9%) (Figure 3). Of the patients with EVT in the anterior circulation and ASPECTS > 6, 38.2% achieved a good outcome (mRS 0–2) compared with 20.2% with ASPECTS <7. Details of risk factors for unfortunate outcomes are described in the Supplementary Material.

## Discussion

The major purpose of our registry was to analyze our in-house management of patients admitted for EVT of acute ischemic stroke. We identified several factors that caused a delay in endovascular treatment. Straightforward patient management was impeded by mandatory medical treatment of a hypertensive crisis, epileptic fits, vomiting, or agitation of the patient. However, the analysis also revealed flaws in the workflow that could be easily addressed.

Despite consensus on the standard procedure, the ER neurologist did not get advance notice of the stroke patient from the emergency medical service in 17% of the cases—a factor that could be easily addressed by reporting to the physician in charge. In approximately one-third of the cases, a delay in the neuroradiological diagnosis and treatment was documented. In several cases, door-to-groin time was prolonged by request for MRI or more than one imaging technique, which was observed more often in patients with unknown time of symptom onset and with less experienced interventionalists. Another less frequent cause of delay was the lack of immediate availability of CCT or MRI.

Imaging protocols in stroke patients differ significantly. A recently published survey including 50 interventional sites from different countries showed that multimodal CT (not contrast-enhanced CT, CTA, and CT perfusion) was the most frequently used imaging modality on admission (58%), followed by not contrast-enhanced CT plus CTA (32%) and multimodal MRI (12%) (18).

In the THRACE trial, where centers were free to use CT or MRI before randomization, CCT needed significantly less time than MRI (19). Accordingly, Kim and colleagues observed an ~25-min delay if MRI was used for the evaluation of acute ischemic stroke patients for EVT compared with CCT (3).

Therefore, MRI should be used only to clarify non-standard cases. Current American Heart Association guidelines (20) recommend that patients with acute ischemic stroke within 6 h of last known normal, LVO, and ASPECTS ≥ 6 be selected for mechanical thrombectomy on the basis of CT and CTA or MRI and MRA. Additional imaging is reserved for patients with wake-up stroke. We caution against excluding patients from EVT by additional imaging studies that are not indicated. Our results emphasize that additional imaging is time-consuming. Because the benefit of EVT decreases with increasing time from symptom onset, strict adherence to standard operating procedures is imperative.

Standardization of stroke care workflow, continuous hospital staff education, and discussion of possible improvements efficiently reduces door-to-recanalization times and improves patient outcome (21). By implementing a dedicated program, Hassan et al. were able to reduce the door-to-recanalization time by ~30% (21).

In patients who are transferred for EVT, the so-called door-in-door-out time at the referring center has been shown to be significantly related to clinical outcome (22). Rapid access of the patient to an EVT center is desirable. In our cohort, onset-to-groin time differed in median 110 min between those patients who were directly admitted and those who were transferred for EVT, and onset-to-recanalization differed by 120 min in median. Approximately one-third of the patients were transferred from hospitals located within a radius of <30 km, most of them in a radius of <10 km. Even in these cases, the transfer took in median 60 min. This loss of time could have been avoided by direct admittance to the EVT center. On-site triage based on the severity of stroke and allocation to an EVT center has been repeatedly recommended to reduce the onset-to-treatment times but is only reluctantly accepted (23–25). Centralization of EVT in centers that are available 24/7 would help to standardize the pre- and intra-hospital management of these patients and facilitate treatment by highly experienced personnel.

**Figure 4:**
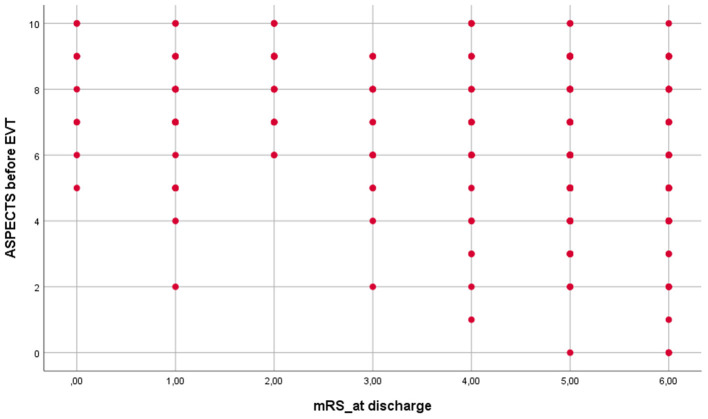
Modified Rankin Scale (mRS) at discharge in relation to Alberta Stroke Program Early CT Score (ASPECTS) on admission (Spearman rho correlation: *r* = −0.295; *p* < 0.001).

In addition to workflow, treatment outcomes were also analyzed. Patients treated at our hospital differed significantly from those included in the 2015 thrombectomy trials (1–5). However, they were quite similar to those presented in the German Stroke Registry Endovascular Treatment, except for the frequency of atrial fibrillation, unknown symptom onset, i.v.-thrombolysis, and premedication with anticoagulants, which all were higher in our sample (7). Nevertheless, the outcome was comparable. At discharge from inpatient rehabilitation (in median 68.5 days after stroke), mortality (30.5 vs. 29%) was similar in our patients, whereas good outcome (28.9 vs. 37%) was less frequent. The latter may be due to the difference in median ASPECTS on admission (9 vs. 7). Of note, both the German Stroke Registry Endovascular Treatment and our single-center registry indicate that patients with ASPECTS ≤ 6 can achieve a favorable outcome (26) (Figure 4). This was also shown by a recent meta-analysis of seven randomized EVT trials (27).

## Conclusion

EVT has been used successfully to treat LVO for more than 5 years, but there is room for improvement both in the prehospital setting and in the hospital. Although our data were collected monocentrically, our results are likely applicable to other hospitals. We believe that consistent and repeated process analysis is critical to further optimize EVT outcomes.

## Data Availability Statement

The raw data supporting the conclusions of this article will be made available by the authors, upon reasonable request.

## Ethics Statement

Ethical review and approval was not required for the study in accordance with the local legislation and institutional requirements. Written informed consent for participation was not required for this study in accordance with the national legislation and the institutional requirements.

## Author Contributions

KW: conceptualization, methodology, data monitoring, data analysis, original draft preparation, draft review, and editing. SG: data acquisition, data analysis, draft review, and editing. GG and MG: data monitoring, draft review, and editing. RS and HW: draft review and editing. OA-F: data acquisition, original draft preparation, draft review, and editing. FG: conceptualization, methodology, original draft preparation, draft review, and editing. All authors contributed to the article and approved the submitted version.

## Conflict of Interest

The authors declare that the research was conducted in the absence of any commercial or financial relationships that could be construed as a potential conflict of interest.

## References

[1] Berkhemer OA Fransen PS Beumer D van den Berg LA Lingsma HF Yoo AJ. A randomized trial of intraarterial treatment for acute ischemic stroke. N Engl J Med. (2015) 372:11–20. 10.1056/NEJMoa1411587 25517348

[2] Goyal M Demchuk AM Menon BK Eesa M Rempel JL Thornton J. Randomized assessment of rapid endovascular treatment of ischemic stroke. N Engl J Med. (2015) 372:1019–30. 10.1056/NEJMoa1414905 25671798

[3] Saver JL Goyal M Bonafe A Diener HC Levy EI Pereira VM. Stent-retriever thrombectomy after intravenous t-PA vs. t-PA alone in stroke. N Engl J Med. (2015) 372:2285–95. 10.1056/NEJMoa1415061 25882376

[4] Campbell BC Mitchell PJ Kleinig TJ Dewey H Churilov L Yassi N. Endovascular therapy for ischemic stroke with perfusion-imaging selection. N Engl J Med. (2015) 372:1009–18. 10.1056/NEJMoa1414792 25671797

[5] Jovin TG Chamorro A Cobo E de Miquel MA Molina CA Rovira A. Thrombectomy within 8 hours after symptom onset in ischemic stroke. N Engl J Med. (2015) 372:2296–306. 10.1056/NEJMoa1503780 25882510

[6] Goyal M Menon BK van Zwam WH Dippel DW Mitchell PJ Demchuk AM. Endovascular thrombectomy after large-vessel ischaemic stroke: a meta-analysis of individual patient data from five randomised trials. Lancet. (2016) 387:1723–31. 10.1016/S0140-6736(16)00163-X 26898852

[7] Wollenweber FA Tiedt S Alegiani A Alber B Bangard C Berrouschot J. Functional outcome following stroke thrombectomy in clinical practice. Stroke. (2019) 50:2500–6. 10.1161/STROKEAHA.119.027415 31337298

[8] Kim JT Cho BH Choi KH Park MS Kim BJ Park JM. Magnetic resonance imaging versus computed tomography angiography based selection for endovascular therapy in patients with acute ischemic stroke. Stroke. (2019) 50:365–72. 10.1161/STROKEAHA.119.025173 30612537

[9] Nogueira RG Jadhav AP Haussen DC Bonafe A Budzik RF Bhuva P. Thrombectomy 6 to 24 hours after stroke with a mismatch between deficit and infarct. N Engl J Med. (2018) 378:11–21. 10.1056/NEJMoa1706442 29129157

[10] Albers GW Marks MP Kemp S Christensen S Tsai JP Ortega-Gutierrez S. Thrombectomy for stroke at 6 to 16 hours with selection by perfusion imaging. N Engl J Med. (2018) 378:708–18. 10.1056/NEJMoa1713973 29364767 PMC6590673

[11] Maingard J Foo M Chandra RV Leslie-Mazwi TM. Endovascular treatment of acute ischemic stroke. Curr Treat Options Cardiovasc Med. (2019) 21:89. 10.1007/s11936-019-0781-9 31823080

[12] Ospel J Kappelhof M Groot AE LeCouffe NE Coutinho JM Yoo AJ. Combined effect of age and baseline alberta stroke program early computed tomography score on post-thrombectomy clinical outcomes in the MR CLEAN registry. Stroke. (2020) 51:3742–5. 10.1161/STROKEAHA.120.031773 33092478

[13] Yoo AJ Berkhemer OA Fransen PSS van den Berg LA Beumer D Lingsma HF. Effect of baseline alberta stroke program early CT score on safety and efficacy of intra-arterial treatment: a subgroup analysis of a randomised phase 3 trial (MR CLEAN). Lancet Neurol. (2016) 15:685–94. 10.1016/S1474-4422(16)00124-1 27302238

[14] Dargazanli C Arquizan C Gory B Consoli A Labreuche J Redjem H. Mechanical thrombectomy for minor and mild stroke patients harboring large vessel occlusion in the anterior circulation: a multicenter cohort study. Stroke. (2017) 48:3274–81. 10.1161/STROKEAHA.117.018113 29089458

[15] Akuttherapie des Ischämischen Schlaganfalls –Ergänzung 2015–Rekanalisierende Therapie –Leitlinien für Diagnostik und Therapie in der Neurologie. DGN (2016).

[16] Puetz V Sylaja PN Coutts SB Hill MD Dzialowski I Mueller P. Extent of hypoattenuation on CT angiography source images predicts functional outcome in patients with basilar artery occlusion. Stroke. (2008) 39:2485–90. 10.1161/STROKEAHA.107.511162 18617663

[17] Zaidat OO Yoo AJ Khatri P Tomsick TA von Kummer R Saver JL. Recommendations on angiographic revascularization grading standards for acute ischemic stroke: a consensus statement. Stroke. (2013) 44:2650–63). 10.1161/STROKEAHA.113.001972 23920012 PMC4160883

[18] Velasco González A Buerke B Görlich D Chapot R Smagge L Velasco MDV. Variability in the decision-making process of acute ischemic stroke in difficult clinical and radiological constellations: analysis based on a cross-sectional interview-administered stroke questionnaire. Eur Radiol. (2019) 29:6275–84 10.1007/s00330-019-06199-4 31076863

[19] Provost C Soudant M Legrand L Ben Hassen W Xie Y Soize S. Magnetic resonance imaging or computed tomography before treatment in acute ischemic stroke. Stroke. (2019) 50:659–64. 10.1161/STROKEAHA.118.023882 30744542

[20] Powers WJ Rabinstein AA Ackerson T Adeoye OM Bambakidis NC Becker K. 2018 Guidelines for the early management of acute ischemic stroke: a guideline for healthcare professionals from the American heart association/American stroke association. Stroke. (2019) 50:e344–418 10.1161/STR.0000000000000158 31662037

[21] Hassan AE Rabah RR Preston L Tekle WG. STEPS-T program improves endovascular treatment outcomes of acute ischemic stroke; a 6-year study. Front Neurol. (2020) 10:1251. 10.3389/fneur.2019.01251 32116978 PMC7029425

[22] McTaggart RA Moldovan K Oliver LA Dibiasio EL Baird GL Hemendinger ML. Door-in-Door-Out time at primary stroke centers may predict outcome for emergent large vessel occlusion patients. Stroke. (2018) 49:2969–74. 10.1161/STROKEAHA.118.021936 30571428

[23] Schlemm L Endres M Nolte CH. Bypassing the closest stroke center for thrombectomy candidates: what additional delay to thrombolysis is acceptable? Stroke. (2020) 51:867–75. 10.1161/STROKEAHA.119.027512 31964288

[24] McTaggart RA Holodinsky JK Ospel JM Cheung AK Manning NW Wenderoth JD. Leaving no large vessel occlusion stroke behind. Reorganizing stroke systems of care to improve timely access to endovascular therapy. Stroke. (2020) 51:1951–60. 10.1161/STROKEAHA.119.026735 32568640

[25] Alexandrov AW Fassbender K. Triage based on preclinical scores-low-cost strategy for accelerating time to thrombectomy. JAMA Neurol. (2020) 77:681–2. 10.1001/jamaneurol.2020.0113 32250425

[26] Deb-Chatterji M Pinnschmidt H Flottmann F Leischner H Broocks G Alegiani A. Predictors of independent outcome of thrombectomy in stroke patients with large baseline infarcts in clinical practice: a multicenter analysis. J Neurointerv Surg. (2020) 12:1064–8. 10.1136/neurintsurg-2019-015641 32107288

[27] Román LS Menon BK Blasco J Hernández-Pérez M Dávalos A Majoie CBLM. Imaging features and safety and efficacy of endovascular stroke treatment: a meta-analysis of individual patient-level data. Lancet Neurol. (2018) 17:895–904. 10.1016/S1474-4422(18)30242-4 30264728

