# Supplementary material for: Endovascular Treatment of Acute Ischemic Stroke in Clinical Practice: Analysis of Workflow and Outcome in a Tertiary Care Center
Source: Front Neurol. 2021 Jun 7;12:657345. doi: 10.3389/fneur.2021.657345 (PMC8215500; doi:10.3389/fneur.2021.657345)
Supplement: Supplementary file 1 [file Table_1.pdf]

Supplementary Table 1

Characteristics of patients with fortunate (mRS 0-2) or unfortunate outcome (mRS 3-5, mRS 6) until discharge

	Patients with mRS 0-2 n=79	Patients with mRS 3-5 n=142	Patients with mRS 6 n=61	p-values * All groups ¶ mRS 0-2 vs mRS 6 § mRS 3-5 vs mRS 6 € mRS 0-2 vs 3-5
Age (median;years) ($25^{th}/75^{th}$ percentile)	71 (64;81)	75 (63;81.25)	81 (72.5;87)	<0.001*¶§€
Sex (m/f)	47/32	71/71	30/31	0.34
Family status Single/married/unknown	23/52/4	54/80/8	60/27/4	0.16
Statutory/private health insurance	56/23	112/30	51/10	0.18
NIHSS on admission	12 (7;16)	16 (12;19)	19 (13;24.5)	<0.001 *¶€ § =0.002
symptom onset unknown	22 (27.8%)	59 (41.5%)	34 (55.7%)	*0.004 ¶ 0.001 € 0.043 § 0.063
intravenous thrombolysis	57 (72%)	104 (73.2%)	43 (70.5%)	0.92
diabetes mellitus	21 (26.6%)	30 (21.1 %)	21 (34.4%)	0.13
hypertension	58 (73.4%)	103 (72.5%)	48 (78.7%)	0.65
hypercholesterolemia	49/78 (62.8%)	64/123 (52.0%)	24/32 (75%)	* <0.043 ¶ 0.22 € 0.13 § 0.02
atrial fibrillation	45/79 (57%)	71/137 (51.8%)	40/44 (90.9%)	* <0.001 ¶ <0.001 § <0.001 € 0.47
Platelet inhibitors	20/79 (25.3%)	42/142 (29.6%)	19/61 (31.1%)	*0.71
Vitamin K antagonist	5/79 (6.3%)	18/142 (12.7%)	14/61 (22.9 %)	*0.015 ¶ 0.004 € 0.14 § 0.065
DOAC/LMWH	13/79 (16.5%)	17/142 (12.0%)	10/61 (16.4%)	0.56
Onset to recanalization (min) (25th;75th percentile)(n=169)	296.5 (208; 374)	297.5 (235;357.5)	325 (222;388)	0.25
ASPECTS (n=227)	8 (7;9)	5(6;8)	6 (4;8)	* 0.002 € 0.003 ¶ 0.009

				§ 0.43
pc ASPECTS (n=36)	9 (7;10)	7 (6;10)	7.5 (4.0;8.75)	* 0.27

*p-value for nonparametric test for k independent samples; € p-value for comparison of mRS 0-2 and mRS 3-5; ¶ p-value for comparison of mRS 0-2 and mRS 6, § p-value for comparison of mRS 3-5 and mRS 6

ASPECTS is less reliable in patients who present within 90 minutes of stroke symptom onset

mRS: modified Rankin Scale; m: male; f: female; NIHSS: National Institute of Health Stroke Scale; DOAC: direct oral anticoagulants; LMWH: low molecular weight heparin; ASPECTS: Alberta Stroke Program Early CT Score ; pc: posterior circulation
