# Supplementary material for: Endovascular Treatment of Acute Ischemic Stroke in Clinical Practice: Analysis of Workflow and Outcome in a Tertiary Care Center
Source: Front Neurol. 2021 Jun 7;12:657345. doi: 10.3389/fneur.2021.657345 (PMC8215500; doi:10.3389/fneur.2021.657345)
Supplement: Supplementary file 2 [file Data_Sheet_1.pdf]

Supplementary results

Frequency of intracranial hemorrhage after EVT

All but 8 patients received a post-interventional CCT. Pooling of contrast medium was detected in 167 cases (61.6%) and ICH in 45 patients (16%): 21 patients suffered secondary parenchymal bleeding (7.4%), 15 subarachnoid hemorrhage (5.3 %), 8 both (2.8%) and 1 a subdural hematoma in addition to the parenchymal bleeding. Eight of the 21 patients with parenchymal bleeding had an increase in their NIHSS of more than 4 points. Intracranial bleeding was more frequent in patients with contrast medium pooling (OR: 3.323; CI: 1.478 – 7.472).

Comparison of patients with fortunate (mRS 0-2) and unfortunate functional outcome (mRS 3-5 and mRS 6)

Patients with good functional outcome were younger, had a lower NIHSS on admission and more often a known time of symptom onset (Suppl. Table 1). Atrial fibrillation was more frequent in the patients who died than in the other patient groups. Onset to recanalization time was not significantly different between the three outcome groups. Multinomial logistic regression analysis was performed to look for factors that increased the chance for good outcome separately for patients with LVO in the anterior or posterior circulation. For patients with LVO in the anterior circulation the chance to achieve mRS 0-2 decreased with increasing NIHSS on admission (mRS 3-5 vs. mRS 0-2; OR 1.148; CI: 1.064 – 1.238, p<0.001) and increased with increasing ASPECTS (mRS 3-5 vs. mRS 0-2; OR: 0.713; CI: 0.583 – 0.871, p=0.001). Patients with TICI 3 were significantly less at risk for a bad outcome than patients with incomplete recanalization (OR: 0.282; CI: 0.133 – 0.596; p=0.001). The risk to die depended in addition significantly on age (OR: 1.098; CI: 1.048 - 1.152; p<0.001). For LVO in the posterior circulation outcome depended on NIHSS on admission (OR: 1.171; CI: 1.024 – 1.339; p=0.021), and successful recanalization decreased the mortality rate significantly (OR: 0.012; CI: 0.000 – 0.372; p=0.012). Similar results were achieved looking at the mRS after the end of in-patient rehabilitation. Of note, a high ASPECTS before EVT does not exclude a fatal outcome, nor does an ASPECTS ≤ 6 exclude a good outcome in the individual case (figure 4).
